# Screening for Suspected Microplastic-like Particles in Post-Mortem Human Liver: Correlating Histopathological Alterations with Clinical Profiles

**DOI:** 10.3390/life16081315

**Published:** 2026-08-11

**Authors:** Eugen-Toma Radu, Nastaca Alina Palade, Crina Cristina Solomon, Oana Gabriela Somcutian, Manuela Pia Pumnea, Maria Totan, Claudia Maria Mihuț, Cecilia Georgescu, Adina Frum, Carmen Maximiliana Dobrea, Felicia Gabriela Gligor

**Affiliations:** 1 County Emergency Clinical Hospital Sibiu, 550025 Sibiu, Romania; eugen.radu@ulbsibiu.ro; 2 Preclinical Department, Faculty of Medicine, Lucian Blaga University of Sibiu, 550169 Sibiu, Romania; manuela-pia.pumnea@ulbsibiu.ro (M.P.P.); maria.totan@ulbsibiu.ro (M.T.); adina.frum@ulbsibiu.ro (A.F.); carmen.dobrea@ulbsibiu.ro (C.M.D.); felicia.gligor@ulbsibiu.ro (F.G.G.); 3 Department of Pathology, “Niculae Stancioiu” Heart Institute Cluj-Napoca, 19–21, Calea Moților St., 400001 Cluj-Napoca, Romania; oanatarta15@yahoo.com; 4 Biology and Ecology Research Center, Faculty of Sciences, Lucian Blaga University of Sibiu, 550012 Sibiu, Romania; claudia.mihut@ulbsibiu.ro; 5 Faculty of Agriculture Sciences, Food Industry and Environmental Protection, Lucian Blaga University of Sibiu, Dr. Ion Rațiu Str. 7–9, 550012 Sibiu, Romania; cecilia.georgescu@ulbsibiu.ro

**Keywords:** microplastics, liver, human tissue, hepatic fibrosis, inflammation, polarized light microscopy, post-mortem study

## Abstract

**Background/Objectives**: Microplastics (MPs) are emerging environmental contaminants with potential implications for human health. Experimental studies suggest that MPs accumulate in the liver and promote inflammatory and fibrotic changes, but evidence from human tissues remains limited. This study investigated the presence of suspected MP-like particles in post-mortem human liver tissue and their associations with clinical, biochemical, hematological and histopathological parameters. **Methods**: Post-mortem liver tissue samples were collected from 55 adults. Suspected MP-like particles were extracted using hydrogen peroxide digestion, filtration, Nile Red staining and image-based quantification. Histopathological evaluation assessed inflammation, fibrosis, necrosis and fatty liver degeneration. Polarized light microscopy was used as a supportive morphological assessment method. Statistical analyses included Mann–Whitney U tests, Fisher’s exact tests and Spearman correlation analysis. **Results**: Detectable hepatic suspected MP-like particles were identified in 9 of 55 individuals (16.4%). Individuals with detectable suspected MP-like particles had significantly lower alanine aminotransferase (ALT) levels and leukocyte counts. The hepatic suspected MP-like particle burden showed weak positive correlation with liver fibrosis and inflammation and weak inverse correlation with ALT levels and leukocyte count. Fisher’s exact test showed that liver fibrosis and liver inflammation were significantly associated with detectable hepatic suspected MP-like particles, with higher unadjusted odds observed in the corresponding 2 × 2 contingency tables. No statistically significant associations were found for liver necrosis or fatty liver degeneration. **Conclusions**: Detectable suspected MP-like particles were identified in post-mortem human liver tissues and were more closely associated with fibrotic and inflammatory histopathological changes than with routine biochemical abnormalities. Larger studies using standardized detection methods are needed to confirm these results.

## 1. Introduction

In recent years, the environmental accumulation of plastics and their degradation into microplastics (MPs) have raised significant concerns regarding potential human exposure [1,2,3,4,5]. Secondary MPs—derived from the mechanical, photochemical and chemical weathering of everyday plastic items—represent the predominant fraction of environmental debris [6,7]. Human uptake occurs daily via ingestion, inhalation, dermal contact and potentially parenteral routes [8,9,10,11,12,13]. Following exposure, the gut–liver axis has been proposed as one of the principal pathways through which translocated particles and absorbed xenobiotics may reach the liver via the portal circulation [14,15,16,17].

Physicochemically, MPs consist of diverse polymer matrices (e.g., polyethylene, polypropylene, polystyrene, polyvinyl chloride) that do not act merely as inert substrates [18,19]. They frequently harbor functional chemical additives, such as phthalates and bisphenol A, while adsorbing ambient contaminants, including heavy metals and persistent organic pollutants [11,20,21]. In experimental in vitro and in vivo models, exposure to these particles has been associated with biological changes, with hepatotoxicity, including inflammatory response, disturbances in lipid metabolism, fibrotic remodeling and hepatocyte apoptosis [22,23]. Whether these findings translate directly to human liver pathology remains uncertain.

However, translating these experimental findings to human liver pathology remains a major challenge. While recent exploratory studies have identified MP fragments within human tissues—with reported sizes ranging from sub-micron particles up to several hundred micrometers [24,25]—the available evidence regarding hepatotoxicity remains largely restricted to preclinical models. Consequently, the hepatic clearance, spatial distribution and long-term histological impact of exogenous microparticles on the human liver parenchyma remain poorly defined.

To address this critical knowledge gap, the present study investigates the presence and spatial distribution of suspected MP-like particles in post-mortem human liver tissues and evaluates their associated tissue-level alterations. By combining histopathological scoring—specifically the Ishak modified Hepatic Activity Index (mHAI) and cross-polarized light microscopy—with ante-mortem clinical and biochemical data, this study aims to investigate the presence of suspected MP-like particles in post-mortem human liver tissue and to explore their associations with histopathological alterations and clinical correlates [26,27].

## 2. Materials and Methods

### 2.1. Study Population

This study used clinical and histopathological data from adult individuals registered at the Sibiu County Emergency Clinical Hospital, Romania, between March 2022 and December 2025. Since liver biopsy is an invasive procedure, a post-mortem study design was chosen. The study was approved by the Ethics Committee of Lucian Blaga University of Sibiu (Approval No. 10/20.07.2021). In total, post-mortem liver tissue samples were collected from 55 individuals of both sexes, aged between 21 and 94 years. Baseline clinical, biochemical, hematological, and histopathological characteristics were evaluated in the overall cohort and according to hepatic MP detection status. The individuals died from various medical conditions that were not considered to be directly related to microplastic exposure.

Written informed consent for the use of post-mortem tissue samples and associated clinical data was obtained from the next of kin for all included cases. However, detailed information regarding living habits, occupational exposure and lifestyle factors was unavailable due to ethical restrictions and the retrospective post-mortem nature of the study.

### 2.2. Data Sources and Variables

Data were retrieved from hospital medical records. The following baseline characteristics were recorded: age, sex, area of residence (urban/rural), presence of neoplastic disease, and history of radiotherapy and/or chemotherapy exposure. Laboratory data included the most recent results available before death, including liver function tests such as alanine aminotransferase (ALT), aspartate aminotransferase (AST), alkaline phosphatase (ALP), and bilirubin levels, as well as metabolic (amylase, lipase), kidney (urea, creatinine, eGFR), electrolyte (sodium, potassium, chloride) and inflammatory (C-reactive protein CRP, procalcitonin) parameters.

### 2.3. Sample Collection for Digestion

For suspected MP-like particle analysis, 5 g of liver tissue was collected in sterile 50 mL tubes with aluminum caps. The use of plastic instruments or instruments containing plastic components was avoided throughout the sampling procedure to reduce the risk of external contamination. The collected samples were preserved in a freezer at −80 °C.

### 2.4. Sample Collection for Histological Analysis

Liver tissue samples were collected within the first 24 h after death from individuals who underwent autopsy to establish the cause of death. After dissection, three tissue fragments of approximately 1 cm^3^ each were collected from the region of interest and fixed in 30 mL of Davidson’s solution in 50 mL capped tubes. Davidson’s solution consisted of 2% formaldehyde solution (concentration of 37–40%), 35% absolute ethyl alcohol, 10% glacial acetic acid, and 53% distilled water, as previously described by Costa (2017) [28]. To minimize the risk of contamination, only instruments that did not contain plastic components were used. Each sample was labeled to ensure traceability and to allow correlation with the corresponding medical records.

### 2.5. Identification and Quantification of Suspected MP-like Particles

Prior to sample processing, all laboratory instruments and glassware were thoroughly rinsed with acetone to minimize the risk of contamination. Strict contamination control measures were implemented throughout the analytical procedure. Laboratory personnel wore cotton laboratory coats, all sample handling was performed using glass materials, and extractions were performed inside a microbiological safety cabinet in an isolated environment without active air circulation. Glass tubes were covered with aluminum foil during incubation to reduce potential airborne contamination.

For each specimen, 1 g of liver tissue was transferred into a 50 mL glass tube followed by the addition of 20 mL of 30% hydrogen peroxide. Samples were incubated at 40 °C for seven days, with periodic vortexing to facilitate tissue digestion. Following incubation, samples were allowed to cool to room temperature and centrifuged at 2000 rpm for two minutes. The resulting supernatants were vacuum-filtered through 1 μm glass-fiber filters. The filters were subsequently dried at 40 °C and treated with an additional 3 mL of 30% hydrogen peroxide at the same temperature to remove residual organic material, rinsed with 40 mL of ultrapure water and dried again in an oven at 40 °C.

To facilitate fluorescence-based detection, approximately 1 mL of Nile Red solution (0.001 mg/mL) was applied to completely cover the filter surface and incubated for 15 min at room temperature. The filters were subsequently rinsed with 60 mL of ultrapure water, dried overnight at 40 °C and examined using a transilluminator (Clare Chemical, Sibiu, Romania). Images were acquired under standardized conditions using the following camera (Canon EOS M6 Mark II, Sibiu, Romania) settings: ISO 200, aperture F6.3, exposure time 1/2 s, and focal length 45 mm.

Procedural quality-control measures were implemented for every analytical batch. A procedural reagent blank was processed in parallel with the tissue samples through the complete digestion, filtration, staining, imaging, and image-analysis workflow. No suspected particles were detected in any blank; therefore, no blank correction was applied to the tissue-sample counts. Method performance was additionally assessed using three recovery controls per analytical batch by spiking each control with ten artificially generated ABS microplastic particles before processing. The recovery controls were subjected to the same digestion, filtration, Nile Red staining, imaging, and counting procedure as the study samples. Recovery was calculated as (number of particles recovered/number of particles added) × 100. Recoveries were 8/10 (80%), 9/10 (90%), and 11/10 (110%), yielding a mean recovery of 93.3%. The minFeret of the ABS particles ranged from 112.74 to 863.65 µm. Recoveries exceeding 100% may be explained by the potential fragmentation of manually generated ABS particles during sample processing, combined with the counting variability and image-analysis uncertainty inherent to measurements based on low particle numbers. These controls supported the ability of the workflow to recover plastic particles while also indicating the measurement variability expected at low particle counts.

Quantification of suspected MP-like particles was performed according to the methodology described by Curtean-Bănăduc et al. (2023) [29], using ImageJ software (version 1.54p). Because the analytical workflow relied on fluorescence staining, image analysis and morphological assessment, without polymer-specific spectroscopic confirmation, the detected structures are referred to throughout the manuscript as suspected MP-like particles.

The entire filter surface was systematically examined. Prior to automated image analysis, all fluorescence images were visually inspected to identify candidate particles based on predefined morphological characteristics, including particle shape, well-defined margins, fluorescence intensity, optical appearance and color. Following ImageJ processing, all automatically detected particles were re-evaluated manually to ensure that they did not correspond to residual Nile Red deposits or other non-specific fluorescent artefacts. Only particles fulfilling both the predefined morphological criteria and the post-processing verification were retained for quantitative analysis.

To reduce background noise and minimize false-positive detection, an operational minimum-size threshold was applied to the minimum Feret diameter (minFeret). Following spatial calibration in ImageJ, the image scale was expressed as *P* pixels/mm. The minimum accepted dimension was set at three pixels; therefore, the corresponding physical threshold was calculated as 3/*P* mm, resulting in a minFeret threshold of 0.05621 mm (56.21 µm). Candidate structures with a minFeret below 56.21 µm were excluded. Structures with minFeret values immediately above this threshold were also excluded when manual review demonstrated repetitive morphology or optical characteristics consistent with background artefacts. This threshold represents an operational image-analysis detection limit specific to the present screening workflow and should not be interpreted as an instrument-specific analytical limit of detection established using polymer-specific chemical measurements.

The present analytical workflow was designed as a screening approach for the detection of suspected MP-like particles based on fluorescence and morphological characteristics. Although this methodology enables standardized particle detection and quantification, it does not provide polymer-specific chemical identification. Consequently, definitive confirmation of particle composition would require complementary analytical techniques such as micro-FTIR, Raman spectroscopy or pyrolysis-gas chromatograph/mass spectrometry.

### 2.6. Histological Assessment

To evaluate liver tissue alterations—including inflammation, fibrosis, necrosis, and steatosis—sections were stained with hematoxylin and eosin (H&E), the gold standard in histopathology [30]. Slides were initially examined under unpolarized light using a Leica (Wetzlar, Germany) DM3000 microscope. For histological scoring, we selected the Ishak modified Hepatic Activity Index (mHAI) over simpler systems such as METAVIR. The Ishak system provides superior histological resolution by evaluating fibrosis on an expanded 7-point scale (0–6) and grading necroinflammatory activity across four independent components, including isolated portal inflammation [26]. For statistical analysis, histopathological findings were dichotomized into binary variables. Fibrosis and inflammation were considered absent when the Ishak score was 0 and present when the Ishak score was ≥1. Necrosis and fatty liver degeneration were also recorded as binary variables (absent/present) based on their histopathological identification.

For the histological visualization of suspected MP-like particles, hematoxylin and eosin (H&E)-stained sections were additionally examined under cross-polarized light using a Leica DM2500 microscope equipped with polarizing filters and an integrated digital camera. While polarized light microscopy primarily aimed to detect exogenous plastic-like microparticles—a technique successfully utilized for over seven decades to identify particle matter in tissue specimens [27]—it also effectively delineated fibrosis by highlighting the intrinsic birefringence of collagen fibers [31,32]. Polarized light microscopy was used as a supportive morphological assessment method to aid in the evaluation of suspected microplastic-like particles and to differentiate them from endogenous birefringent structures where possible.

All histopathological slides were evaluated independently by two pathologists who were blinded to the fluorescence-based particle-detection results and to the calculated hepatic particle burden. The two assessments were subsequently compared, and any discrepancies in histopathological grading or staging were resolved by joint review and consensus. Thus, the initial evaluations were independent and blinded, whereas the final value entered in the study database represented the consensus assessment.

### 2.7. Suspected MP-like Particle Size Estimation

The size of the identified suspected MP-like particles was estimated using ImageJ software, following a methodology based on Curtean-Bănăduc et al. (2023) [29], together with polarized light microscopy analysis. Particle size measurements were performed on acquired microscopic images after calibration of the image scale. The results were expressed in micrometers (µm).

### 2.8. Statistical Analysis

Statistical analysis was performed using IBM SPSS Statistics version 26.0 (IBM Corp., Armonk, NY, USA). Graphical representations, including correlation heatmaps, raincloud plots, and forest plots, were generated using Python version 3.12.13 (Python Software Foundation, Wilmington, DE, USA), with pandas version 2.2.3 and NumPy version 2.3.5.

Continuous variables were assessed for distribution using graphical inspection and descriptive statistics. Given the non-normal distribution of most variables and the relatively small number of individuals with detectable hepatic suspected MP-like particles, continuous variables were expressed as median and interquartile range (IQR). Categorical variables were summarized as absolute and relative frequencies [n (%)].

Comparisons between individuals without detectable hepatic suspected MP-like particles and those with detectable hepatic suspected MP-like particles were performed using the Mann–Whitney U test for continuous variables and Fisher’s exact test for categorical variables. Fisher’s exact test was preferred for categorical comparisons because of the small sample size and the presence of low expected cell counts.

Associations between histopathological liver alterations and detectable hepatic suspected MP-like particles were evaluated using 2 × 2 contingency tables. Odds ratios (ORs) with 95% confidence intervals (95% CIs) were calculated as measures of association, and statistical significance was assessed using the two-sided Fisher’s exact test.

Correlation analyses between hepatic suspected MP-like particle burden and clinical, biochemical, hematological, and histopathological parameters were performed using Spearman’s rank correlation coefficient (rho). Correlation strength was interpreted as follows: rho < 0.20, very weak correlation; rho = 0.20–0.39, weak correlation; rho = 0.40–0.59, moderate correlation; rho = 0.60–0.79, strong correlation; and rho ≥ 0.80, very strong correlation. Positive rho values indicated direct correlations, whereas negative rho values indicated inverse correlations.

Missing laboratory values were treated as missing data and excluded pairwise from the corresponding analyses. Because this was an exploratory study with a limited sample size, no formal adjustment for multiple comparisons was applied. Accordingly, the results of the multiple univariable analyses should be interpreted as exploratory and hypothesis-generating rather than confirmatory.

## 3. Results

### 3.1. Study Population Characteristics

Detectable hepatic suspected MP-like particles were identified in 9 of the 55 included individuals (16.4%), whereas 46 individuals (83.6%) were classified as the suspected MP-like particle–absent group (Table 1). No statistically significant differences were observed between the suspected MP-like particle–absent, and suspected MP-like particle–present groups in terms of age, male sex, urban residence, neoplasm prevalence, exposure to radiotherapy and/or chemotherapy, direct bilirubin (DBIL), or total bilirubin (TBIL).

Regarding liver biomarkers, ALT levels were significantly lower in the suspected MP-like particle–present group compared with the suspected MP-like particle–absent group [15.00 vs. 23.00 U/L, *p* = 0.026], whereas AST levels did not differ significantly between groups. No statistically significant differences were observed in pancreatic and renal biomarkers, electrolyte parameters, or hematological variables, except for leukocyte count. Leukocyte counts were significantly lower in the suspected MP-like particle–present group compared with the suspected MP-like particle–absent group [8.54 vs. 14.41, *p* = 0.018]. Because multiple clinical and laboratory variables were evaluated, these findings should be interpreted as exploratory. Inflammatory biomarkers, including C-reactive protein (CRP) and procalcitonin, did not differ significantly between groups.

### 3.2. Distribution of Hepatic Suspected MP-like Particle Burden Among Suspected MP-like Particle–Present Cases

**Figure 1:**
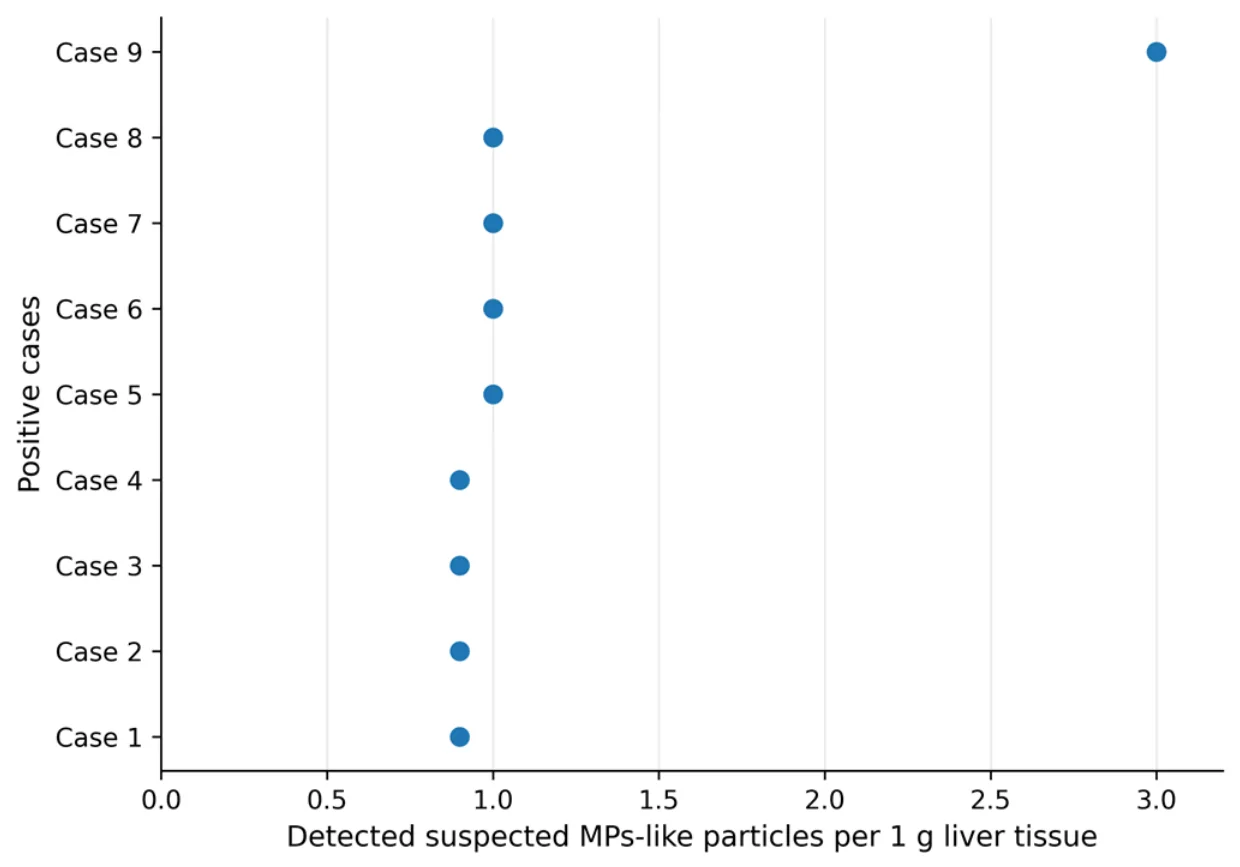
Distribution of hepatic suspected MP-like particle burden among individuals in the suspected MP-like particle–present group. Most patients (P1–P8) exhibited a similar hepatic suspected MP-like particle burden (approximately 0.9–1.0/g), whereas patient 9 (P9) showed a markedly higher suspected MP-like particle burden (3.0/g), indicating substantially greater hepatic suspected MP-like particle accumulation compared with the remainder of the cohort.

Among the nine screening-positive cases, the hepatic suspected MP-like particle burden was low in most specimens. For each specimen, burden was calculated as the integer number of retained candidate particles divided by the exact mass of liver tissue processed (g) and was therefore expressed as particles/g. Although approximately 1 g of tissue was targeted, the measured specimen mass varied by up to ±0.002 g. This minor variation produced negligible differences after normalization: detection of one retained candidate particle corresponded to approximately 0.998–1.002 particles/g and was reported as 1.0 particle/g after rounding to one decimal place. Eight cases therefore had a burden of 1.0 particle/g, whereas one case had a burden of 3.0 particles/g (Figure 1). These values represent mass-normalized particle-count densities and should not be interpreted as fractional particle counts or arbitrary units.

### 3.3. Biomarker Differences According to Hepatic Suspected MP-like Particle Detection Status

**Figure 2:**
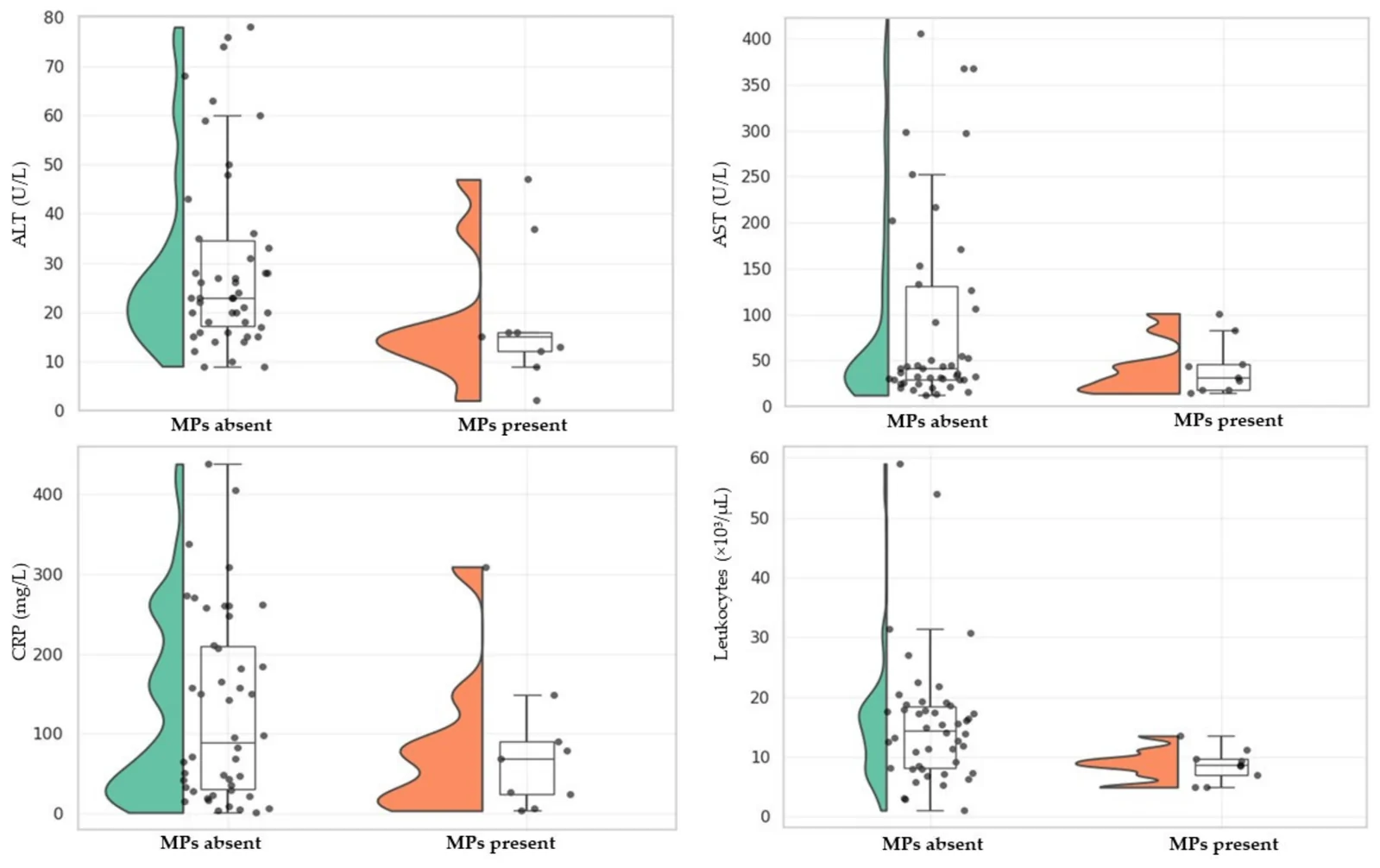
Distribution of liver injury and inflammatory biomarkers according to hepatic suspected MP-like particle detection status.

Raincloud plots showed the distribution of liver and inflammatory biomarkers according to hepatic suspected MP-like particle detection status (Figure 2). For ALT, individuals in the suspected MP-like particle–present group showed lower values and a narrower distribution compared with the suspected MP-like particle–absent group, in agreement with the statistically significant difference observed in Table 1. AST values also appeared generally lower in the suspected MP-like particle–present group; however, the distributions overlapped, and the between-group difference was not statistically significant.

Regarding inflammatory biomarkers, leukocyte counts were lower and less variable in the suspected MP-like particle–present group compared with the suspected MP-like particle–absent group, consistent with the statistically significant difference reported in Table 1. In contrast, CRP values showed wide variability in both groups, without a clear visual separation between the suspected MP-like particle–absent and suspected MP-like particle–present groups. Overall, the graphical analysis suggests lower ALT and leukocyte values among individuals with detectable hepatic suspected MP-like particles, whereas AST and CRP did not show a clear separation according to hepatic suspected MP-like particle detection status.

### 3.4. Correlation Between Hepatic Suspected MP-like Particles and Clinical, Biochemical, Hematological, and Histopathological Parameters

**Figure 3:**
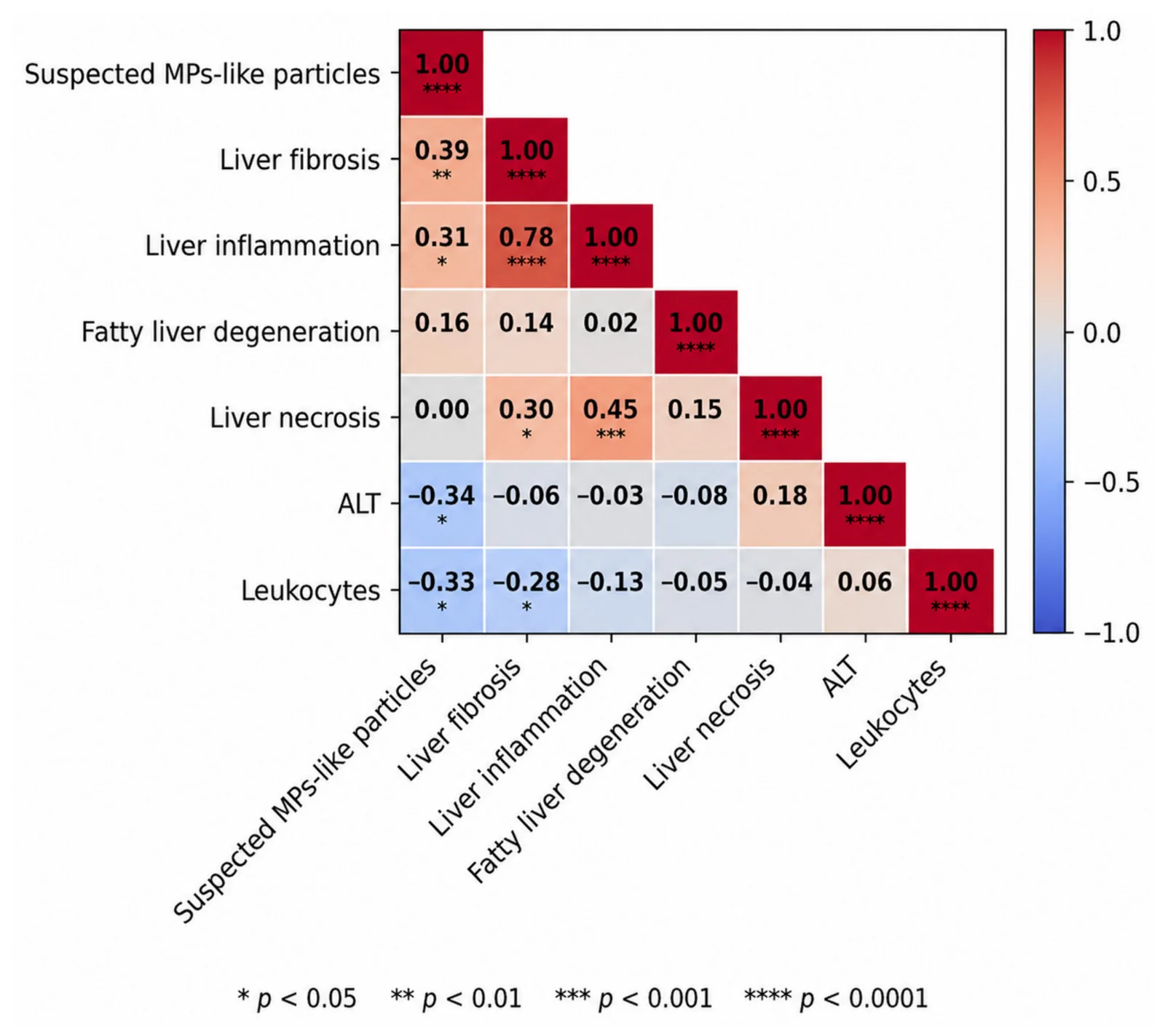
Spearman correlation heatmap of hepatic suspected MP-like particles and selected histopathological and laboratory parameters.

Spearman correlation analysis was performed to evaluate the relationships between hepatic suspected MP-like particle burden and the assessed clinical, biochemical, hematological, and histopathological parameters. The correlations involving the main variables of interest are presented in Figure 3, whereas the complete correlation matrix is provided in Supplementary Figure S1. Hepatic suspected MP-like particle burden showed a weak positive correlation with liver fibrosis (rho = 0.39, *p* < 0.01) and liver inflammation (rho = 0.31, *p* < 0.05), suggesting that higher hepatic suspected MP-like particle burden may be associated with fibrotic and inflammatory liver alterations. In contrast, hepatic suspected MP-like particle burden was weakly and inversely correlated with ALT levels (rho = −0.34, *p* < 0.05) and leukocyte count (rho = −0.33, *p* < 0.05). No statistically significant correlations were observed between hepatic suspected MP-like particle burden and AST, CRP, creatinine, electrolyte parameters, and most other hematological biomarkers (Supplementary Figure S1).

Regarding histopathological alterations, liver inflammation showed a strong positive correlation with liver fibrosis (rho = 0.78, *p* < 0.0001), whereas liver necrosis showed a weak positive correlation with fibrosis (rho = 0.30, *p* < 0.05). Overall, the correlation analysis suggests that hepatic suspected MP-like particle burden was more closely related to inflammatory and fibrotic histopathological alterations than to routine biochemical or hematological parameters.

### 3.5. Histopathological Associations with Detectable Hepatic Suspected MP-like Particles

Associations between histopathological liver alterations and detectable hepatic suspected MP-like particles were evaluated using 2 × 2 contingency tables. Because of the small sample size and the presence of low cell counts, statistical significance was assessed using the two-sided Fisher’s exact test. Odds ratios (ORs) with 95% confidence intervals (CIs) were calculated as measures of association (Table 2). Because only nine individuals had detectable hepatic suspected MP-like particles, the estimated odds ratios should be interpreted cautiously.

Liver fibrosis was significantly associated with detectable hepatic suspected MP-like particles (OR = 9.917, 95% CI: 1.805–54.485; *p* = 0.003). Similarly, liver inflammation showed a significant association with hepatic suspected MP-like particle detection (OR = 6.563, 95% CI: 1.218–35.371; *p* = 0.017). In contrast, liver necrosis was not significantly associated with detectable hepatic suspected MP-like particles (OR = 0.833, 95% CI: 0.088–7.898; *p* = 0.874). Fatty liver degeneration also showed no statistically significant association with hepatic suspected MP-like particle detection (OR = 2.344, 95% CI: 0.551–9.972; *p* = 0.241).

Overall, detectable hepatic suspected MP-like particles were more frequently observed in individuals with fibrotic and inflammatory histopathological liver alterations, whereas necrosis and fatty liver degeneration did not show statistically significant associations. However, the confidence intervals around the estimated odds ratios were wide, reflecting the limited number of positive cases. Therefore, these findings should be interpreted as exploratory and require confirmation in larger studies.

### 3.6. Exploratory Associations Between Selected Clinical Diagnoses and Detectable Hepatic Suspected MP-like Particles

Exploratory analyses of selected diagnoses according to hepatic suspected MP-like particle detection status are presented in Table 3. No statistically significant associations were observed between detectable hepatic suspected MP-like particles and any of the evaluated diagnoses. Any cardiovascular disease was less frequent in the suspected MP-like particle–present group than in the suspected MP-like particle–absent group, but this difference was not significant (66.7% vs. 82.6%, *p* = 0.362). Atherosclerosis showed a similar distribution between groups (33.3% vs. 32.6%, *p* = 1.000). Myocardial infarction was numerically more frequent in the suspected MP-like particle–present group (33.3% vs. 17.4%), although the association was not statistically significant (*p* = 0.362). Bronchopneumonia also showed no difference between groups (44.4% vs. 43.5%, *p* = 1.000). Overall, these exploratory findings did not indicate a clear association between detectable hepatic suspected MP-like particles and the selected cardiovascular or pulmonary diagnoses.

### 3.7. Microscopic Visualization of Suspected Hepatic MP-like Particles Under Polarized Light

**Figure 4:**
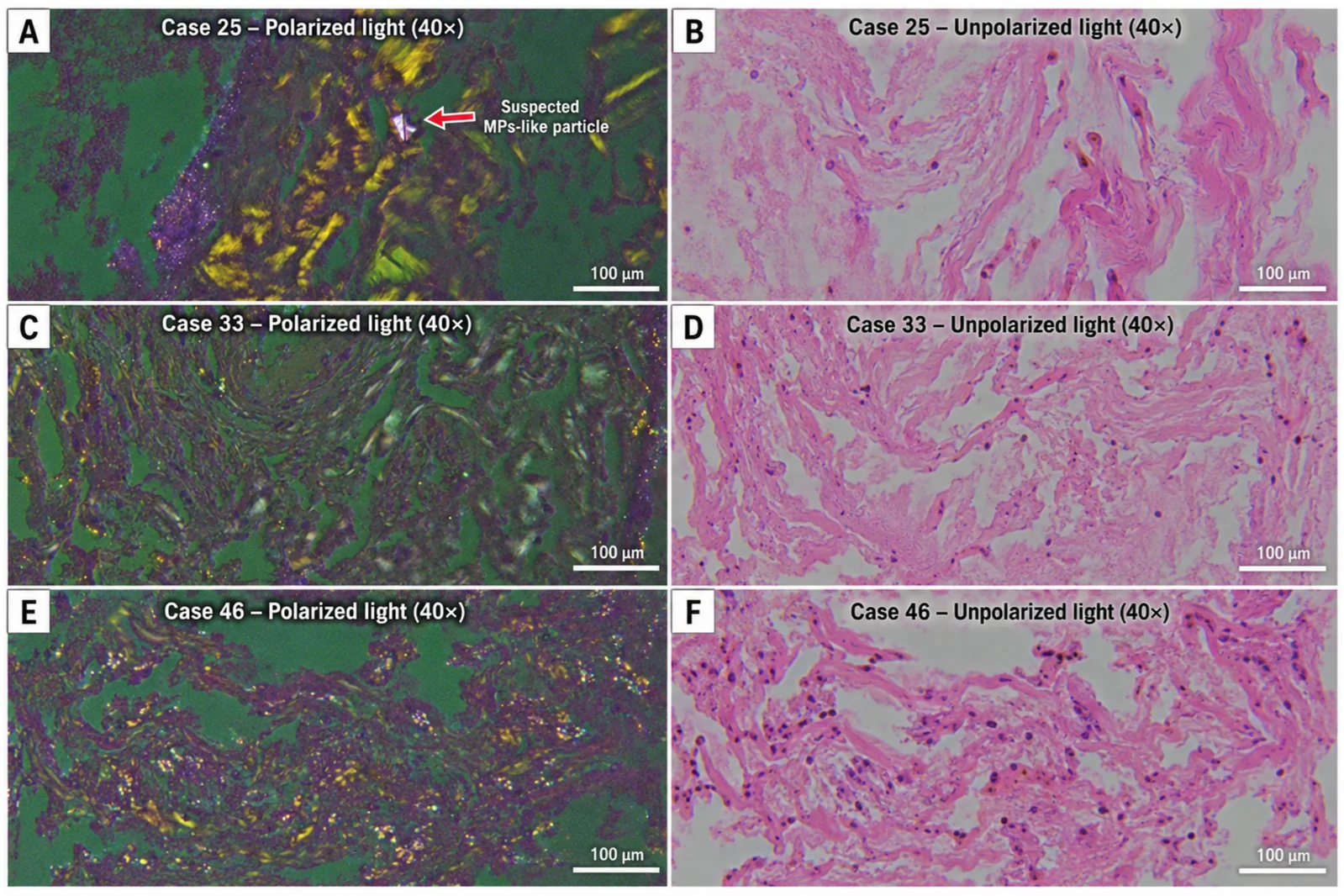
Representative polarized and unpolarized light microscopy images of liver tissue. (**A**,**B**) Case 25: suspected MP-like birefringent particle observed under polarized light and the corresponding unpolarized field. (**C**,**D**) Case 33: birefringent collagen fibers and cholesterol-like particles, with no suspected MP-like particles, and the corresponding unpolarized field. (**E**,**F**) Case 46: birefringent collagen fibers and cholesterol-like particles, with no suspected MP-like particles, and the corresponding unpolarized field. The colors observed in the polarized-light images result from birefringence under polarized illumination and do not represent specific histological staining. Magnification: 40×. Scale bar: 100 μm. MPs, microplastic.

Polarized light microscopy was used as a supportive morphological assessment method to evaluate suspected MP-like particles in representative liver sections (Figure 4). Paired polarized and unpolarized images were analyzed in three selected cases to compare suspected synthetic particles with endogenous birefringent structures, including collagen fibers and cholesterol-like particles. In case 25 (Figure 4A,B), a discrete birefringent structure suspected to correspond to a plastic microparticle was observed within a fibrotic portal area. This structure showed a regular geometric appearance, well-defined margins, sharp edges, and distinct purple-white birefringence under polarized light, remaining clearly separated from the surrounding collagen fibers. In cases 33 (Figure 4C,D) and 46 (Figure 4E,F), polarized light microscopy revealed birefringent collagen fibers and cholesterol-like particles, but no discrete suspected MP-like particles with similar morphology were observed. Collagen fibers appeared as irregular, tissue-integrated structures with variable thickness and yellowish birefringence.

Overall, these representative images illustrate the morphological and optical criteria used to differentiate suspected MP-like particles from endogenous birefringent tissue components. However, polarized light microscopy was considered a supportive visual approach and not a standalone confirmatory method for polymer identification.

## 4. Discussion

This post-mortem study investigated the presence of detectable hepatic suspected MP-like particles in human liver tissue and their relationship with clinical, biochemical, hematological and histopathological parameters. Detectable hepatic suspected MP-like particles were identified in a minority of the included individuals, while the main associations were observed with histopathological liver alterations, particularly fibrosis and inflammation. These findings are in line with previous human tissue-based evidence showing that suspected MP-like particles can be detected in liver tissue, particularly in individuals with underlying liver disease [24]. In contrast, most routine biochemical, inflammatory, renal, electrolyte and hematological parameters did not show clear differences according to hepatic suspected MP-like particle detection status.

Both Spearman correlation analysis and the 2 × 2 contingency table analysis indicated that the detection of hepatic suspected MP-like particles was more strongly associated with liver fibrosis and inflammation than with necrosis or fatty liver degeneration. These findings are consistent with experimental and toxicological evidence suggesting that suspected MP-like particles may induce particle-related toxicity, oxidative stress, inflammatory responses and tissue remodeling [13,22,30]. However, given the post-mortem and observational design of the study, these associations should not be interpreted as evidence of causality. The interpretation of these findings should also take into account the marked clinical heterogeneity of the study population. A substantial proportion of individuals had neoplastic diseases and had undergone chemotherapy or radiotherapy, while others presented with terminal illness, infectious conditions, and pre-existing liver disease or were receiving multiple medications. These factors may independently influence liver histopathology and laboratory biomarkers and therefore represent potential confounders. Because only nine individuals had detectable hepatic suspected MP-like particles, multivariable adjustment for these variables was not statistically feasible. Consequently, the reported associations should be interpreted with appropriate caution and require confirmation in larger, more homogeneous study populations.

Regarding routine laboratory parameters, ALT levels and leukocyte counts were lower in the suspected MP-like particles, whereas AST, CRP, procalcitonin, renal biomarkers, electrolytes and most hematological parameters did not differ significantly between groups. These findings suggest that routine biochemical markers may not reliably reflect the presence of detectable hepatic suspected MPs—like particles or localized histopathological alterations. However, because only nine individuals were classified as positive and multiple clinical and laboratory variables were evaluated without formal adjustment for multiple comparisons, the observed differences in ALT and leukocyte counts may represent chance findings but should be interpreted with caution. Accordingly, these results should be regarded as hypothesis-generating rather than confirmatory and require validation in larger independent cohorts. The discrepancy between tissue-level changes and conventional laboratory biomarkers underscores the need for future studies exploring more sensitive indicators of particle-related tissue injury, local inflammation, oxidative stress and fibrotic remodeling [18,30]. Given the small number of positive cases and the post-mortem nature of the cohort, these findings may have been influenced by underlying clinical conditions, terminal physiological status, treatment exposure, the interval between the last laboratory assessment and death, and the multiple statistical comparisons performed. Therefore, these observations should regarded as hypothesis-generating rather than confirmatory and require validation in larger, independent cohorts.

Exploratory analyses of selected clinical diagnoses did not identify significant associations between detectable hepatic suspected MP-like particles and cardiovascular or pulmonary diagnoses. Although myocardial infarction was numerically more frequent among individuals with detectable hepatic suspected MP-like particles, the difference was not statistically significant, and the confidence intervals were wide. Therefore, these findings should be interpreted as hypothesis-generating and not as evidence of a disease-specific association.

Polarized light microscopy was used as a supportive morphological assessment method approach for suspected MP-like particles in representative liver sections. In one case, a discrete birefringent structure with regular morphology, sharp margins, and distinct optical behavior was observed within a fibrotic portal area. In contrast, other representative cases showed birefringent collagen fibers and cholesterol-like particles without similar discrete suspected MP-like particles. These observations illustrate the usefulness of polarized light microscopy for morphological assessment and differentiation from endogenous birefringent structures, as previously suggested in experimental settings [33]. Nevertheless, this method cannot provide definitive polymer identification or distinguish synthetic MPs from other exogenous birefringent material [24,30]. Therefore, the observations should be interpreted as morphological evidence only; definitive characterization would require complementary analytical techniques capable of determining particle chemical composition.

The liver is a biologically plausible site for MP accumulation due to its anatomical and functional connection with the gastrointestinal tract through portal circulation. After ingestion, MPs may cross the intestinal barrier and reach the liver through the portal vein, intestinal lymphatic system or systemic circulation [18,30]. Experimental studies have suggested that particle size, surface properties and chemical composition may influence uptake, translocation and tissue accumulation [13,22]. However, in the present study, the route of exposure and translocation could not be directly determined.

This study has several limitations. First, the number of individuals with detectable hepatic suspected MP-like particles was small, which limits statistical power and increases uncertainty around effect estimates. Second, the post-mortem retrospective design does not allow causal inference or assessment of temporal relationships. Third, the study population was clinically heterogeneous, including individuals with neoplastic diseases, previous chemotherapy or radiotherapy, terminal illness, infectious conditions, and other comorbidities, all of which may have influenced liver histopathology and laboratory findings independently of suspected MP-like particles. Because of the limited number of positive cases, adjustment for these potential confounding factors was not statistically feasible. In addition, information regarding lifestyle, occupational exposure, dietary habits, medication use, and environmental exposure was unavailable. Fourth, polarized light microscopy was used as a supportive visualization method, but polymer-specific confirmation was not performed. Finally, missing laboratory values for some biomarkers limited the interpretation of selected analyses.

Despite these limitations, the study provides preliminary human tissue-based evidence that detectable hepatic suspected MP-like particles may be associated with localized histopathological alterations, particularly fibrosis and inflammation. These findings support the need for larger studies using standardized contamination-control procedures, quantitative MP assessment and polymer-specific identification methods to clarify the potential role of MPs in human liver pathology. Finally, multiple exploratory statistical comparisons were performed without formal adjustment for multiple testing. Therefore, some statistically significant findings may represent false-positive associations and should be interpreted cautiously.

## 5. Conclusions

This exploratory post-mortem study identified suspected hepatic MP-like particles in a subset of human liver tissue samples and demonstrated preliminary associations with histopathological evidence of liver fibrosis and inflammation. In contrast, most routine biochemical and hematological parameters did not differ significantly between samples with and without detectable suspected microplastic-like particles, suggesting that localized tissue alterations may not be reflected by conventional circulating biomarkers under the conditions of this study.

Given the absence of polymer-specific analytical confirmation and the observational design of the study, these findings should be interpreted with caution and should not be considered evidence of causality. Nevertheless, they provide preliminary human tissue-based evidence supporting further investigation into the potential relationship between suspected MP-like particles and liver pathology.

Further studies incorporating standardized contamination-control procedures, polymer-specific identification techniques and larger, well-characterized cohorts are warranted to validate these observations and clarify the biological and clinical significance of suspected MP-like particles in the human liver.

## Figures and Tables

**Table 1:** Baseline clinical, biochemical, and hematological characteristics of the study population according to hepatic microplastic detection status.

Variable	Overall (*n* = 55)	Suspected MP-like Particles Absent (*n* = 46)	Suspected MP-like Particles Present (*n* = 9)	*p*-Value
**Demographic characteristics**				
Age, years	68.00 (59.50, 82.00) (*n* = 55)	66.50 (57.00, 81.00) (*n* = 46)	73.00 (62.00, 86.00) (*n* = 9)	0.393
Male sex, n (%)	29 (52.7) (*n* = 55)	23 (50.0) (*n* = 46)	6 (66.7) (*n* = 9)	0.360
Urban residence, n (%)	34 (61.8) (*n* = 55)	29 (63.0) (*n* = 46)	5 (55.6) (*n* = 9)	0.672
**Clinical characteristics**				
Neoplasm, n (%)	41 (74.5) (*n* = 55)	36 (78.3) (*n* = 46)	5 (55.6) (*n* = 9)	0.153
Radiotherapy and/or chemotherapy, n (%)	47 (85.5) (*n* = 55)	41 (89.1) (*n* = 46)	6 (66.7) (*n* = 9)	0.080
Blood glucose, mg/dL	132.00 (102.00, 194.00) (*n* = 55)	130.50 (89.00, 193.00) (*n* = 46)	143.00 (118.00, 195.00) (*n* = 9)	0.474
**Liver biomarkers**				
DBIL, mg/dL	0.94 (0.81, 1.30) (*n* = 15)	0.94 (0.82, 1.30) (*n* = 11)	0.915 (0.79, 1.30) (*n* = 4)	0.240
TBIL, mg/dL	0.81 (0.56, 1.19) (*n* = 55)	0.78 (0.55, 1.12) (*n* = 46)	1.08 (0.73, 1.26) (*n* = 9)	0.460
AST, U/L	41.00 (28.50, 103.50) (*n* = 55)	41.00 (29.00, 133.00) (*n* = 46)	31.00 (18.00, 46.00) (*n* = 9)	0.202
ALT, U/L	23.00 (15.50, 34.00) (*n* = 55)	23.00 (17.00, 35.00) (*n* = 46)	15.00 (12.00, 16.00) (*n* = 9)	0.026
**Pancreatic biomarkers**				
Amylase, U/L	49.00 (33.00, 75.00) (*n* = 55)	49.50 (33.00, 82.00) (*n* = 46)	42.00 (34.00, 63.00) (*n* = 9)	0.585
Lipase, U/L	27.00 (16.50, 105.50) (*n* = 16)	34.00 (16.00, 105.50) (*n* = 12)	27.00 (21.00, 72.50) (*n* = 4)	1.000
**Renal biomarkers**				
Urea, mg/dL	54.00 (40.50, 89.00) (*n* = 55)	51.00 (40.00, 87.00) (*n* = 46)	84.00 (52.00, 92.00) (*n* = 9)	0.311
Creatinine, mg/dL	1.26 (0.80, 2.06) (*n* = 55)	1.27 (0.80, 2.18) (*n* = 46)	1.15 (0.80, 1.63) (*n* = 9)	0.776
eGFR, mL/min/1.73 m^2^	58.00 (29.00, 86.00) (*n* = 55)	56.50 (27.00, 91.00) (*n* = 46)	71.00 (31.00, 84.00) (*n* = 9)	0.856
**Electrolytes**				
Sodium, mmol/L	139.00 (136.00, 143.50) (*n* = 55)	139.00 (136.00, 142.00) (*n* = 46)	144.00 (135.00, 144.00) (*n* = 9)	0.657
Potassium, mmol/L	4.28 (3.52, 4.86) (*n* = 55)	4.31 (3.80, 4.88) (*n* = 46)	3.42 (3.40, 4.70) (*n* = 9)	0.446
Chloride, mmol/L	102.00 (99.00, 107.00) (*n* = 55)	103.35 (99.00, 107.00) (*n* = 46)	99.00 (97.00, 107.00) (*n* = 9)	0.327
**Inflammatory biomarkers**				
C-reactive protein (CRP), mg/L	79.50 (27.40, 195.67) (*n* = 55)	89.18 (29.70, 210.43) (*n* = 46)	68.30 (24.20, 90.50) (*n* = 9)	0.275
Procalcitonin, ng/mL	1.58 (0.32, 33.39) (*n* = 14)	1.18 (0.31, 17.98) (*n* = 12)	28.10 (1.42, 54.77) (*n* = 2)	0.440
**Haematological biomarkers**				
Leukocytes, ×10^3^/µL	12.64 (8.03, 17.71) (*n* = 55)	14.41 (8.04, 18.56) (*n* = 46)	8.54 (6.97, 9.65) (*n* = 9)	0.018
Erythrocytes, ×10^6^/µL	4.06 (3.39, 4.73) (*n* = 55)	4.13 (3.39, 4.79) (*n* = 46)	3.55 (3.14, 4.34) (*n* = 9)	0.280
Platelets, ×10^3^/µL	218.00 (132.00, 313.00) (*n* = 55)	222.50 (152.00, 324.00) (*n* = 46)	141.00 (101.00, 218.00) (*n* = 9)	0.139
Hemoglobin, g/dL	12.10 (10.50, 14.00) (*n* = 55)	12.25 (10.30, 14.00) (*n* = 46)	11.00 (9.40, 13.30) (*n* = 9)	0.453
Hematocrit, %	36.90 (30.70, 41.75) (*n* = 55)	37.05 (31.40, 42.10) (*n* = 46)	32.60 (25.00, 39.50) (*n* = 9)	0.369

Note: Data are presented as median (IQR) for continuous variables and as n (%) for categorical variables. The number of available observations is reported in parentheses when missing data were present. *p*-values refer to comparisons between the suspected MP-like particle–absent and suspected MP-like particle–present groups and were calculated using the Mann–Whitney U test for continuous variables and Fisher’s exact test for categorical variables. IQR, interquartile range; DBIL, direct bilirubin; TBIL, total bilirubin; AST, aspartate aminotransferase; ALT, alanine aminotransferase; eGFR, estimated glomerular filtration rate; CRP, C-reactive protein.

**Table 2:** Histopathological alterations according to hepatic suspected MP-like particle detection.

Histopathological Alteration	Suspected MP-like Particles Absent (*n* = 46)	Suspected MP-like Particles Present (*n* = 9)	OR (95% CI)	*p*-Value
Fibrosis	12 (26.1%)	7 (77.8%)	9.917 (1.805–54.485)	0.003
Inflammation	16 (34.8%)	7 (77.8%)	6.563 (1.218–35.371)	0.017
Necrosis	6 (13.0%)	1 (11.1%)	0.833 (0.088–7.898)	0.874
Fatty liver degeneration	16 (34.8%)	5 (55.6%)	2.344 (0.551–9.972)	0.241

Note: Data are presented as n/N (%). Odds ratios (ORs) and 95% confidence intervals (CIs) were calculated from the raw 2 × 2 contingency tables. Statistical significance was assessed using the two-sided Fisher’s exact test.

**Table 3:** Exploratory associations between selected clinical diagnoses and detectable hepatic microplastics.

Diagnosis	Overall (*n* = 55)	Suspected MP-like Particles Absent (*n* = 46)	Suspected MP-like Particles Present (*n* = 9)	*p*-Value	OR (95% CI)
Any cardiovascular disease	44 (80.0)	38 (82.6)	6 (66.7)	0.362	0.42 (0.09–2.05)
Atherosclerosis	18 (32.7)	15 (32.6)	3 (33.3)	1.000	1.03 (0.23–4.71)
Myocardial infarction	11 (20.0)	8 (17.4)	3 (33.3)	0.362	2.38 (0.49–11.55)
Bronchopneumonia	24 (43.6)	20 (43.5)	4 (44.4)	1.000	1.04 (0.25–4.38)

Note: Data are presented as n (%). *p*-values refer to comparisons between the suspected MP-like particle–absent and suspected MP-like particle–present groups and were calculated using Fisher’s exact test. ORs are unadjusted and were calculated from 2 × 2 contingency tables. Any cardiovascular disease was defined as the presence of atherosclerosis, chronic ischemic heart disease, or myocardial infarction. OR, odds ratio; CI, confidence interval.

## Data Availability

The original contributions presented in this study are included in the article. Further inquiries can be directed to the corresponding authors.

## Supplementary Materials

The following supporting information can be downloaded at: https://www.mdpi.com/article/10.3390/life16081315/s1, Figure S1: Complete Spearman correlation matrix of all assessed clinical, biochemical, hematological, and histopathological parameters.
