# Supplementary material for: Screening for Suspected Microplastic-like Particles in Post-Mortem Human Liver: Correlating Histopathological Alterations with Clinical Profiles
Source: Life (Basel). 2026 Aug 11;16(8):1315. doi: 10.3390/life16081315 (PMC13514284; doi:10.3390/life16081315)
Supplement: Supplementary file 1 [file life-16-01315-s001.zip › life-4451031-supplementary.pdf]

Supplementary

Spearman Correlation Heatmap of Hepatic MPs and Clinical Parameters

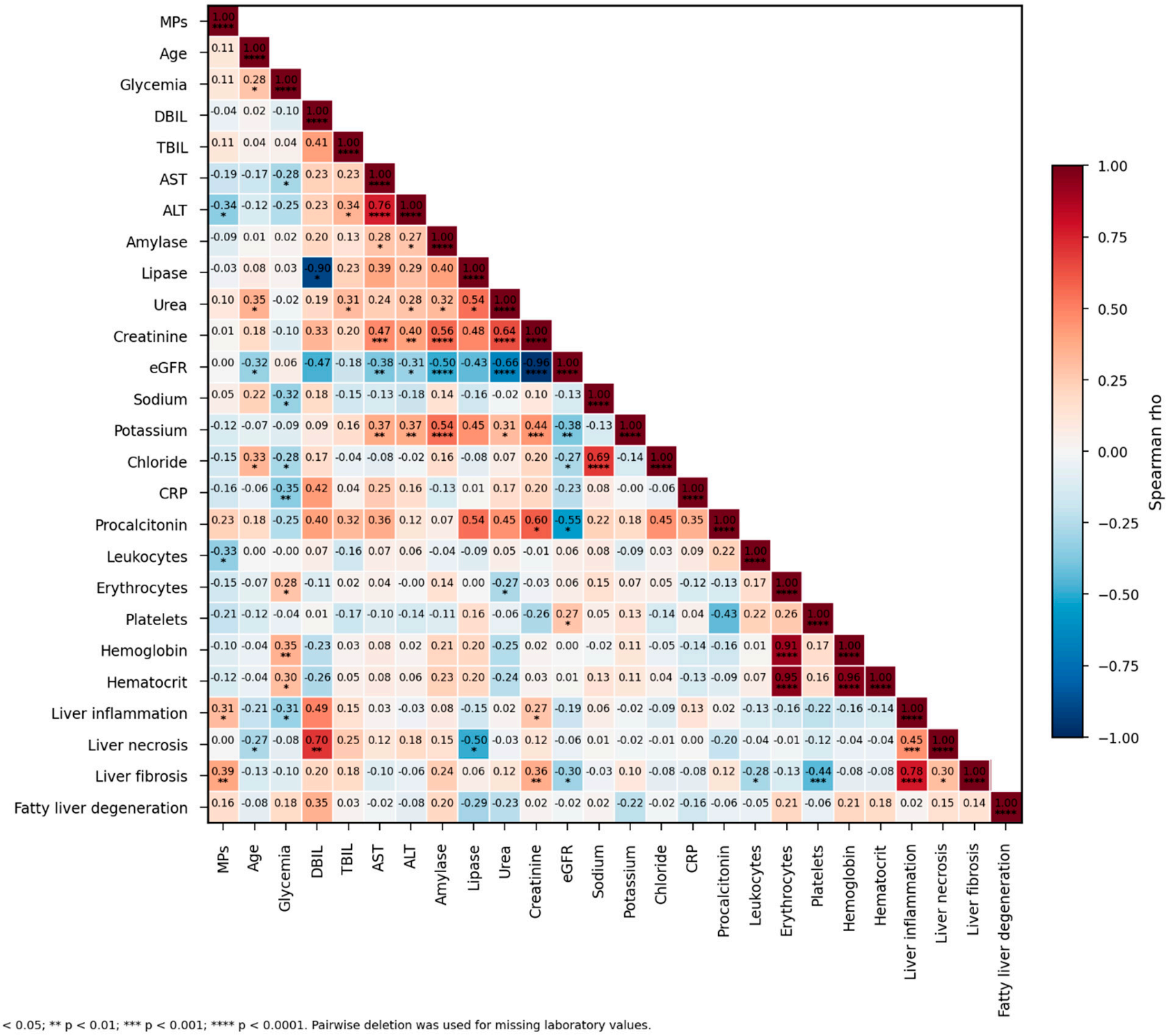

* $p < 0.05$; ** $p < 0.01$; *** $p < 0.001$; **** $p < 0.0001$. Pairwise deletion was used for missing laboratory values.

Supplementary Figure S1. Complete Spearman correlation matrix of all assessed clinical, biochemical, hematological, and histopathological parameters
